# Existence of reservoir with finite-dimensional output for universal reservoir computing

**DOI:** 10.1038/s41598-024-56742-7

**Published:** 2024-04-11

**Authors:** Shuhei Sugiura, Ryo Ariizumi, Toru Asai, Shun-ichi Azuma

**Affiliations:** 1 Graduate School of Engineering, Nagoya University, Nagoya, 464-8603 Japan; 2 Department of Mechanical Systems Engineering, Tokyo University of Agriculture and Technology, Koganei, Tokyo 184-8588 Japan; 3 Graduate School of Informatics, Kyoto University, Kyoto, 606-8501 Japan

**Keywords:** Machine learning, Neural network, Nonlinear dynamical system, Reservoir computing, Applied mathematics, Computational science

## Abstract

In this paper, we prove the existence of a reservoir that has a finite-dimensional output and makes the reservoir computing model universal. Reservoir computing is a method for dynamical system approximation that trains the static part of a model but fixes the dynamical part called the reservoir. Hence, reservoir computing has the advantage of training models with a low computational cost. Moreover, fixed reservoirs can be implemented as physical systems. Such reservoirs have attracted attention in terms of computation speed and energy consumption. The universality of a reservoir computing model is its ability to approximate an arbitrary system with arbitrary accuracy. Two sufficient reservoir conditions to make the model universal have been proposed. The first is the combination of fading memory and the separation property. The second is the neighborhood separation property, which we proposed recently. To date, it has been unknown whether a reservoir with a finite-dimensional output can satisfy these conditions. In this study, we prove that no reservoir with a finite-dimensional output satisfies the former condition. By contrast, we propose a single output reservoir that satisfies the latter condition. This implies that, for any dimension, a reservoir making the model universal exists with the output of that specified dimension. These results clarify the practical importance of our proposed conditions.

## Introduction

Reservoir computing (RC) is a machine learning method for dynamical system approximation. An RC model consists of a dynamical system called a reservoir and a static function called a readout. First, the input signal to the RC model is processed in the reservoir. Next, the signal from the reservoir is processed using the readout, and the model output is obtained. The concept of RC is that only the static part of the model is trained, that is, the readout, to make the model behave as desired^1–5^. RC was proposed initially to simplify the training of recurrent neural networks (RNNs)^6,7^ and is superior in terms of computational cost for training. Generally, a randomly generated RNN is used as the reservoir. However, because the reservoir is fixed, a physical system that is difficult to adjust can also be used as the reservoir. Recently, physical RC, which uses a physical system as the reservoir, has received considerable attention^8–12^. Physical reservoirs are expected to be superior to an RNN implemented on a general-purpose computer in terms of processing speed and energy consumption^8,13^.

We say that an RC model is universal if it can approximate an arbitrary system with arbitrary accuracy by training only the readout. Several studies have been performed on the approximation ability of RC models. Grigoryeva et al.^14^ studied an echo state network (ESN)^1^, which is a typical RC model composed of an RNN reservoir and linear readout. Gonon et al.^15^ studied the three classes of RC models for stochastic inputs: one composed of a linear reservoir and polynomial readout, one composed of a state-affine reservoir and linear readout, and an ESN. They showed that each model can approximate any system with any accuracy. However, their results differ from the universality that we deal with in this paper. This is because, for each target system to be approximated, they must train both the readout and the reservoir. Fixing the reservoir regardless of the targets is the concept of RC, and by violating it, advantages such as the hardware implementation of the reservoir and reduction of the computational cost of training are lost.

We say that a reservoir is universal if it makes the RC model universal. To the best of our knowledge, it is unknown whether a universal reservoir with a finite-dimensional output exists. This is an important problem because a reservoir’s output is finite in practice. The keys to solving this problem are the study of Maass et al.^2^ and our recent study^16^. In each of these studies on continuous-time RC, a sufficient condition for a reservoir to be universal was proposed. They use a polynomial readout, evaluate the approximation error using the uniform norm, and assume that the target has fading memory. Fading memory means that if two inputs were close to each other in the recent past, the present outputs are also close. In^2,16^, an *m*-output reservoir is represented as a set of *m* operators, which are maps between input functions and scalar-valued output functions. Therefore, we call a reservoir with a finite-dimensional output a finite reservoir. In^2^, input and output functions of the operators are defined on the bi-infinite-time (BIT) interval ${\mathbb {R}}$. Hence, we call these operators BIT operators. Maass et al.^2^ showed that a reservoir is universal if it has the separation property and operators in it have fading memory. In^16^, input and output functions of the operators are defined on the right-infinite-time (RIT) interval ${\mathbb {R}}_+=\left[ 0,\infty \right)$. Hence, we call these operators RIT operators. In^16^, we showed that a reservoir is universal if it has the neighborhood separation property (NSP) and the operators in it are bounded. However, it remains an open question whether there exists any finite reservoir satisfying those conditions^2,16^.

In this paper, we provide two results. First, we show that no finite reservoir satisfies the condition in^2^. We derive a contradiction from the assumption that an *m*-output reservoir satisfies the condition in^2^: the separation property and fading memory. BIT operators have a one-to-one correspondence between functionals, which are maps from input signals to ${\mathbb {R}}$. Using the functionals, we construct a map from the compact space of input signals to ${\mathbb {R}}^m$. The separation property and fading memory mean that the constructed map is injective and continuous, respectively. This leads to the contradiction that the space of input signals and a subset of ${\mathbb {R}}^m$ are homeomorphic, although they have different dimensions. As the second result, we show that there is a reservoir that has a single output and the NSP, which is the condition in^16^. RIT operators also have a one-to-one correspondence between functionals. We show that a single output reservoir with the NSP exists if a functional with a continuous left inverse exists. To obtain such a functional, we use the Hahn–Mazurkiewicz theorem, which provides continuous surjection from $\left[ 0,1\right]$ to the space of functional inputs. Assuming the axiom of choice, we can take the right inverse of the surjection, which is the functional that we seek.

Our contribution in this study is to show that there is no finite reservoir satisfying the condition in^2^ but there is one satisfying the condition in^16^. Through the discussion, we provide an example of a universal reservoir with a single output. The mathematical meaning of our example is that an operator, which is a map between functions, can be approximated by training only the readout, which is a continuous map from $\left[ 0,1\right]$ to ${\mathbb {R}}$. This is a counter-intuitive and interesting result. The practical meaning of our example is that a reservoir has the possibility of being universal, regardless of the dimension of the output. This is particularly important for using physical reservoirs because they are difficult to adjust.

The structure of this paper is as follows: first, we show that there is no finite reservoir that satisfies the condition in^2^. Second, we show that a universal reservoir with a single output exists that satisfies the condition in^16^. Finally, we conclude the paper.

## Reservoir computing represented by bi-infinite-time operators

In the study by Maass et al.^2^, which is one of the earliest on RC, they proposed a condition for the reservoir to be universal. We first consider this condition to prove the existence of a universal finite reservoir. Eventually, we conclude that no finite reservoir can satisfy the condition in^2^. In the first subsection, we briefly review^2^, and in the second subsection, we describe one of the two main results of the present study.

### Condition for universality

RC is a method to approximate a dynamical system, a map between functions of time. Let $A\subset {\mathbb {R}}^n$ be a compact set of input values and $K>0$ be the limit of the speed of input change. We define a set $U^B$ of BIT inputs as follows:

1 $$\begin{aligned} U^B = \left\{ u:{\mathbb {R}} \rightarrow A\;\left| \;\forall t_1,t_2 \in {\mathbb {R}},\left\| u{\left( t_1 \right) } - u{\left( t_2 \right) } \right\| \le K \left| {t_1-t_2} \right| \right. \right\} . \end{aligned}$$

Let $Y^B$ be a set of output functions from ${\mathbb {R}}$ to ${\mathbb {R}}$. We call operators from $U^B$ to $Y^B$ “BIT operators” because they are maps between signals defined on the BIT interval ${\mathbb {R}}$. For operator *F* and input signal *u*, we write the output signal and its value at time *t* as *Fu* and $Fu{\left( t\right) }$, respectively. Maass et al.^2^ defined the reservoir, that is, the dynamical part of a RC model, as a set ${\mathbb {F}}$ of BIT operators. If the cardinal number $m=\left| {\mathbb {F}}\right|$ is finite, ${\mathbb {F}}$ represents a dynamical system that returns *m* output signals $F_1u,\ldots ,F_mu\in Y^B$ for an input signal $u\in U^B$. Hence, we call *m* the output dimension of ${\mathbb {F}}$ and call ${\mathbb {F}}$ an *m*-output reservoir. Mathematically, ${\mathbb {F}}$ can even be an uncountable set: for example, the reservoir representing waves on a liquid surface^17^ has the cardinality of continuum.

The RC model ${\hat{F}}:U^B\rightarrow Y^B$ is defined as follows:

2 $$\begin{aligned} {\hat{F}}u{\left( t\right) }=p{\left( F_1u{\left( t\right) },\dots ,F_iu{\left( t\right) }\right) }\quad \left( u\in U^B,t\in {\mathbb {R}}\right) , \end{aligned}$$

where $i\in {\mathbb {N}}$ and $F_1,\dots ,F_i\in {\mathbb {F}}$. The function $p:{\mathbb {R}}^i\rightarrow {\mathbb {R}}$ is a polynomial called a readout. If *m* is finite, we can set $i=m$ and $\left\{ F_1,\dots ,F_i\right\} ={\mathbb {F}}$. In this case, the RC model is trained only by turning the readout *p*. However, if *m* is infinite, we must also select a finite number of operators $F_1,\dots ,F_i\in {\mathbb {F}}$. This operation effectively means the training of the reservoir as well as the readout. For instance, in^18^, they considered a reservoir composed of all operators defined by a stable linear system. In practice, selecting the operators from such a reservoir means the turning of some parameters of a linear system. Therefore, a finite reservoir is required to achieve the low computational cost of training, which is the advantage of RC. In the remainder of this paper, we assume that *m* is finite unless otherwise specified.

The RC model and its reservoir are said to be universal if the model can approximate an arbitrary dynamical system with arbitrary accuracy. We formulate the universality of a reservoir represented by BIT operators as follows:

#### Definition 1

Let reservoir ${\mathbb {F}}$ be a set of BIT operators $F_1,\dots ,F_m$ and let ${\mathbb {F}}^*$ be another set of BIT operators. Reservoir ${\mathbb {F}}$ is said to be universal for uniform approximations in ${\mathbb {F}}^*$ if, for any operator $F^*\in {\mathbb {F}}^*$ and $\varepsilon >0$, a polynomial $p:{{\mathbb {R}}}^m \rightarrow {\mathbb {R}}$ exists that satisfies

3 $$\begin{aligned} \forall u\in U^B,\forall t\in {\mathbb {R}},\left| F^*u{\left( t \right) }-p{\left( F_1u{\left( t \right) },\ldots ,F_mu{\left( t \right) } \right) } \right| <\varepsilon . \end{aligned}$$

Definition 1 means that, if the reservoir ${\mathbb {F}}$ is universal, a polynomial of operators $F_1,\dots ,F_m$ can approximate any target in ${\mathbb {F}}^*$ with any accuracy.

To guarantee the universality of RC, Maass et al.^2^ considered BIT operators that have two properties called time invariance and fading memory. An operator $F:U^B\rightarrow Y^B$ is said to be time-invariant if the following holds:

4 $$\begin{aligned} \forall t\in {\mathbb {R}}, \forall u_1,u_2\in U^B,\left[ \left( \forall \tau \in {\mathbb {R}}, u_2{\left( \tau \right) }=u_1{\left( \tau -t\right) }\right) \Rightarrow \left( \forall \tau \in {\mathbb {R}}, Fu_2{\left( \tau \right) }=Fu_1{\left( \tau -t\right) }\right) \right] . \end{aligned}$$

The property time invariance means that a temporal shift of an input also shifts the output.

Fading memory is defined as follows:

#### Definition 2

A BIT operator $F:U^B\rightarrow Y^B$ is said to have fading memory if the following holds:

5 $$\begin{aligned} \forall u_1\in U^B,\forall \varepsilon>0,\exists \delta>0,\exists T>0,\forall u_2\in U^B,\left[ \max _{\tau \in \left[ -T,0\right] }\left\| u_1{\left( \tau \right) }-u_2{\left( \tau \right) }\right\|<\delta \Rightarrow \left| Fu_1{\left( 0\right) }-Fu_2{\left( 0\right) }\right| <\varepsilon \right] . \end{aligned}$$

Fading memory means that the output value strongly depends on the recent past input but weakly depends on the distant past. Fading memory also means independence from the future, which we define as causality later in the paper.

According to Maass et al.^2^, the following condition is essential for a reservoir to be universal.

#### Definition 3

Let reservoir ${\mathbb {F}}$ be a set of BIT operators. Reservoir ${\mathbb {F}}$ is said to have the separation property if ${\mathbb {F}}$ satisfies the following:

6 $$\begin{aligned} \forall u_1,u_2\in U^B,\exists F\in {\mathbb {F}},\left[ \left( \exists \tau \le 0,u_1{\left( \tau \right) }\ne u_2{\left( \tau \right) }\right) \Rightarrow Fu_1{\left( 0\right) }\ne Fu_2{\left( 0\right) }\right] . \end{aligned}$$

The separation property means that the reservoir provides different outputs to different inputs. Suppose that the reservoir does not have the separation property, that is, the reservoir returns the same output to two different inputs. Then, the RC model cannot approximate a target that returns different outputs to those inputs. Hence, the separation property is necessary to achieve universality. Note that Eqs. (5) and (6) are the conditions for the output at time 0 because time invariance is assumed. Under time invariance, the same holds at times other than 0.

The result in^2^ is described as follows:

#### Theorem 1

(Maass and Natschl^2^) Let ${\mathbb {F}}^*$ be the set of time-invariant BIT operators with fading memory. Suppose that reservoir ${\mathbb {F}}\subset {\mathbb {F}}^*$ has the separation property. Then, reservoir ${\mathbb {F}}$ is universal for uniform approximations in ${\mathbb {F}}^*$.

To summarize the result in^2^, a reservoir with the separation property that contains only time-invariant operators with fading memory is universal. If *m* can be infinite, a reservoir that satisfies the condition of Theorem 1 exists. For example, the reservoir composed of linear systems^18^, which we mentioned before, satisfies the condition.

### No finite reservoir satisfies the condition of Theorem 1

We show that any finite reservoir does not satisfy the condition of Theorem 1. To achieve this, we define a functional corresponding to a time-invariant and causal BIT operator, with which fading memory and the separation property are expressed more simply. The causality of a BIT operator is defined as follows:

#### Definition 4

BIT operator $F:U^B\rightarrow Y^B$ is said to be causal if *F* satisfies

7 $$\begin{aligned} \forall u_1,u_2\in U^B,\forall t\in {\mathbb {R}},\left[ \left( \forall \tau \le t, u_1{\left( \tau \right) }=u_2{\left( \tau \right) }\right) \Rightarrow Fu_1{\left( t\right) }=Fu_2{\left( t\right) }\right] . \end{aligned}$$

A BIT operator with fading memory is causal. With causality, the output value $Fu{\left( t\right) }$ depends only on the input values of *u* on $\left( -\infty ,t\right]$. Moreover, with time invariance, this relation does not depend on *t*. Hence, a causal and time-invariant operator is considered as a relation between input signals on ${\mathbb {R}}_-=\left( -\infty ,0\right]$ and ${\mathbb {R}}$. The corresponding functional is defined by such a relation. For the compact set $A\subset {\mathbb {R}}^n$ and $K>0$ in Eq. (1), we define the domain *V* of functionals as follows:

8 $$\begin{aligned} V = \left\{ v:{\mathbb {R}}_- \rightarrow A\;\left| \;\forall t_1,t_2 \le 0,\left\| v{\left( t_1 \right) } - v{\left( t_2 \right) } \right\| \le K \left| {t_1-t_2} \right| \right. \right\} . \end{aligned}$$

The correspondence between a BIT operator and a functional is defined as follows:

#### Definition 5

A causal and time-invariant BIT operator $F:U^B\rightarrow Y^B$ and a functional $f:V\rightarrow {\mathbb {R}}$ are said to correspond to each other if the following holds:

9 $$\begin{aligned} \forall u\in U^B,\forall v\in V, \left[ \left( \forall \tau \le 0,u{\left( \tau \right) }=v{\left( \tau \right) }\right) \Rightarrow Fu{\left( 0\right) }=f{\left( v\right) }\right] . \end{aligned}$$

This correspondence is one-to-one^18^ and we can express the conditions of operators using their corresponding functionals.

We define a norm on *V* and a metric derived from it. Let $w:{\mathbb {R}}_+\rightarrow \left( 0,1 \right]$ be a non-increasing function that satisfies $\lim _{t\rightarrow \infty }w{\left( t\right) }=0$. We define a weighted norm $\left\| v\right\| _w$ of $v\in V$ as follows:

10 $$\begin{aligned} \left\| v\right\| _w=\sup _{\tau \le 0}\left\| v{\left( \tau \right) }\right\| w{\left( -\tau \right) }. \end{aligned}$$

Using this norm, we define a metric $d:V\times V\rightarrow {\mathbb {R}}_+$ on *V* as follows:

11 $$\begin{aligned} d{\left( v_1,v_2\right) }=\left\| v_1-v_2\right\| _w. \end{aligned}$$

Hereafter, we use *d* as a metric on *V*. Fading memory and the separation property are expressed by a functional as follows:

#### Proposition 1

A causal and time-invariant operator $F:U^B\rightarrow Y^B$ has fading memory if and only if the functional $f:V\rightarrow {\mathbb {R}}$ corresponding to *F* is continuous.

#### Proposition 2

Let $F_1,\ldots ,F_m:U^B\rightarrow Y^B$ be causal and time-invariant operators and functionals $f_1,\ldots ,f_m:V\rightarrow {\mathbb {R}}$ correspond to $F_1,\ldots ,F_m$, respectively. Then, reservoir ${\mathbb {F}}=\left\{ F_1,\ldots ,F_m\right\}$ has the separation property if and only if the following map $\left( f_1,\ldots ,f_m\right) :V\rightarrow {\mathbb {R}}^m$ is injective:

12 $$\begin{aligned} \left( f_1,\ldots ,f_m\right) {\left( v\right) }=\left( f_1{\left( v\right) },\ldots ,f_m{\left( v\right) }\right) \quad \left( v\in V\right) . \end{aligned}$$

We provide the proofs in the supplementary material. Using Propositions 1 and 2, we obtain the following theorem, which provides the conclusion of this section.

#### Theorem 2

Suppose that the input range $A\subset {\mathbb {R}}^n$ in Eq. (1) includes distinct $a_1$ and $a_2$ that satisfy

13 $$\begin{aligned} \forall \alpha \in \left[ 0,1\right] ,a_1+\alpha \left( a_2-a_1\right) \in A \end{aligned}.$$

Then, no natural number *m* and time-invariant operators $F_1,\ldots ,F_m:U^B\rightarrow Y^B$ with fading memory exist such that reservoir ${\mathbb {F}}=\left\{ F_1,\ldots ,F_m\right\}$ has the separation property.

#### Proof of Theorem 2

We prove the theorem by contradiction. Suppose that natural number *m* and time-invariant operators $F_1,\ldots ,F_m:U^B\rightarrow Y^B$ with fading memory exist such that reservoir ${\mathbb {F}}=\left\{ F_1,\ldots ,F_m\right\}$ has the separation property. Let functionals $f_1,\ldots ,f_m:V\rightarrow {\mathbb {R}}$ correspond to $F_1,\ldots ,F_m$, respectively. Then, from Propositions 1 and 2, the map $\left( f_1,\ldots ,f_m\right) :V\rightarrow {\mathbb {R}}^m$ is a continuous injection. An injection can be considered as a bijection onto its image, and a continuous bijection from a compact domain to a Hausdorff space is a homeomorphism. Because the set *V* is compact^16^, map $\left( f_1,\ldots ,f_m\right)$ is a topological embedding, that is, a homeomorphism onto its image. We use the following lemma. $\square$

#### Lemma 1

A topological embedding $g:\left[ 0,1\right] ^{m+1}\rightarrow V$ exists.

We provide the proof after the Proof of Theorem 2. From Lemma 1, the composition $\left( f_1,\ldots ,f_m\right) \circ g:\left[ 0,1\right] ^{m+1}\rightarrow {\mathbb {R}}^m$ is also a topological embedding, that is, $\left[ 0,1\right] ^{m+1}$ and $\left( f_1,\ldots ,f_m\right) \circ g\left( \left[ 0,1\right] ^{m+1}\right) \subset {\mathbb {R}}^m$ are homeomorphic. We call the small inductive dimension simply a dimension. Dimensions have the following two properties (see pages 3–4 of reference^19^): first, two homeomorphic topological spaces have the same dimension. Second, a topological space has a dimension equal to its subspace or larger. Therefore, we have

14 $$\begin{aligned} m+1={\textrm{ind}}\;\left[ 0,1\right] ^{m+1}={\textrm{ind}}\;\left( f_1,\ldots ,f_m\right) \circ g\left( \left[ 0,1\right] ^{m+1}\right) \le {\textrm{ind}}\;{\mathbb {R}}^m=m, \end{aligned}$$

where ind$\left( \cdot \right)$ is the dimension. Eq. (14) is a contradiction, which proves Theorem 2. $\square$

As shown in Theorem 2, any finite reservoir does not satisfy the condition of Theorem 1. Therefore, Theorem 1 cannot support the possibility that a universal finite reservoir exists. We prove Lemma 1 as follows:

#### Proof of Lemma 1

From the assumption, distinct $a_1$, $a_2\in A$ exist that satisfy $a_1+\alpha \left( a_2-a_1\right) \in A$ for any $\alpha \in \left[ 0,1\right]$. Let $T=\frac{1}{K}\left\| a_1-a_2\right\|$. For $c\in \left[ 0,1\right] ^{m+1}$, we define a continuous piecewise linear function $\alpha _c:{\mathbb {R}}_-\rightarrow \left[ 0,1\right]$ as follows:

15 $$\begin{aligned} \alpha _c{\left( t\right) }=c_i+\frac{1}{T}\left( -iT-t\right) \left( c_{i+1}-c_i\right) \quad \left( -\left( i+1\right) T<t\le -iT\right) \end{aligned}.$$

where $i\in \left\{ 0\right\} \cup {\mathbb {N}}$, $\left( c_1,\ldots ,c_{m+1}\right) =c$, and $c_0,c_{m+2},c_{m+3},\ldots$ are zeros. The function $\alpha _c$ satisfies $\alpha _c{\left( -iT\right) }=c_i$. Because the Lipschitz constant of $\alpha _c$ is $\frac{K}{\left\| a_1-a_2\right\| }$ or less, we can define a continuous function $g:\left[ 0,1\right] ^{m+1}\rightarrow V$ as follows:

16 $$\begin{aligned} g:c\mapsto v\quad \left( c\in \left[ 0,1\right] ^{m+1}\right) ,\quad v{\left( t\right) }=a_1+\alpha _c{\left( t\right) }\left( a_2-a_1\right) \quad \left( t\le 0\right) \end{aligned}.$$

Because $v=g{\left( c\right) }$ satisfies $v{\left( -iT\right) }=a_1+c_i\left( a_2-a_1\right)$ for any $c=\left( c_1,\ldots ,c_{m+1}\right) \in \left[ 0,1\right] ^{m+1}$ and $i\in \left\{ 1,\ldots ,m+1\right\}$, *g* is injective. Therefore, *g* is a continuous bijection from its compact domain $\left[ 0,1\right] ^{m+1}$ to its image, that is, *g* is a topological embedding, which proves Lemma 1. $\square$

## Reservoir computing represented by right-infinite-time operators

To obtain the output value of a BIT operator, we have to consider an input signal that continues from the infinite past. In our recent study^16^, we avoided this impracticality by defining input and output signals for positive time and proposed another condition for the reservoir to be universal. Using this condition, we prove the existence of a universal finite reservoir. In the first subsection, we briefly explain our previous results^16^. In the second subsection, we prove the main result of the present study.

### Condition for universality

For compact set $A\subset {\mathbb {R}}^n$ and $K>0$ in Eq. (1), we define a set $U^R$ of RIT inputs as follows:

17 $$\begin{aligned} \begin{aligned} U^R = \left\{ u:{\mathbb {R}}_+ \rightarrow A\;\left| \;\forall t_1,t_2\ge 0,\left\| u{\left( t_1 \right) } - u{\left( t_2 \right) } \right\| \le K \left| {t_1-t_2} \right| \right. \right\} . \end{aligned} \end{aligned}$$

Let $Y^R$ be the set of output functions from ${\mathbb {R}}_+$ to ${\mathbb {R}}$. We call operators from $U^R$ to $Y^R$ “RIT operators” because they are maps between signals defined on the RIT interval ${\mathbb {R}}_+$. In^16^, the reservoir is defined as a set ${\mathbb {F}}$ of RIT operators. The RC model ${\hat{F}}:U^R\rightarrow Y^R$ is defined as follows:

18 $$\begin{aligned} {\hat{F}}u{\left( t\right) }=p{\left( F_1u{\left( t\right) },\dots ,{F_m}u{\left( t\right) }\right) }\quad \left( u\in U^R,t\ge 0\right) , \end{aligned}$$

where $\left\{ F_1,\dots ,F_m\right\} = {\mathbb {F}}$. The polynomial $p:{{\mathbb {R}}^m}\rightarrow {\mathbb {R}}$ is the readout.

In the RIT operator case, universality is defined as follows:

#### Definition 6

Let reservoir ${\mathbb {F}}$ be a set of RIT operators $F_1,\ldots ,F_m$ and let ${\mathbb {F}}^*$ be another set of RIT operators. Reservoir ${\mathbb {F}}$ is said to be universal for uniform approximations in ${\mathbb {F}}^*$ if, for any operator $F^*\in {\mathbb {F}}^*$ and $\varepsilon >0$, a polynomial $p:{{\mathbb {R}}}^m \rightarrow {\mathbb {R}}$ exists that satisfies

19 $$\begin{aligned} \forall u\in U^R,\forall t\ge 0,\left| F^*u{\left( t \right) }-p{\left( F_1u{\left( t \right) },\ldots ,F_mu{\left( t \right) } \right) } \right| <\varepsilon . \end{aligned}$$

RIT operators also correspond to functionals, which are maps to real numbers. We require these corresponding functionals to describe the result in^16^. To correspond to a functional, an RIT operator must be causal as follows:

#### Definition 7

An RIT operator $F:U^R\rightarrow Y^R$ is said to be causal if *F* satisfies

20 $$\begin{aligned} \forall u_1,u_2\in U^R,\forall t\ge 0,\left[ \left( \forall \tau \in \left[ 0,t\right] , u_1{\left( \tau \right) }=u_2{\left( \tau \right) }\right) \Rightarrow Fu_1{\left( t\right) }=Fu_2{\left( t\right) }\right] . \end{aligned}$$

With causality, the output value $Fu{\left( t\right) }$ depends only on the input values of *u* on $\left[ 0,t\right]$. Hence, a causal RIT operator is considered as a relation between input signals of various lengths and ${\mathbb {R}}$. The corresponding functional is defined by such a relation. For the set *V* in Eq. (8), we define the domain $V^{\textrm{res}}$ of functionals as follows:

21 $$\begin{aligned} V^{\textrm{res}} =\left\{ v^{\left[ t \right] }\;\left| \; v \in V, t \ge 0 \right. \right\} , \end{aligned}$$

where $v^{\left[ t \right] }$ is the restriction of *v* to $\left[ -t,0\right]$. Let $\lambda :V^{\textrm{res}}\rightarrow {\mathbb {R}}_+$ be a map defined by $v^{\left[ t \right] }\mapsto t$. For example, $\lambda {\left( v\right) }=1$ for $v:{\left[ -1,0\right] }\rightarrow A$. The correspondence between an RIT operator and a functional is defined as follows:

#### Definition 8

A causal RIT operator $F:U^R\rightarrow Y^R$ and a functional $f:V^{\textrm{res}}\rightarrow {\mathbb {R}}$ are said to correspond to each other if the following holds:

22 $$\begin{aligned} \forall u\in U^R,\forall v\in V^{\textrm{res}},\left[ \left( \forall \tau \in \left[ -t,0\right] ,u{\left( t+\tau \right) }=v{\left( \tau \right) }\right) \Rightarrow Fu{\left( t\right) }=f{\left( v\right) }\right] , \end{aligned}$$

where $$t=\lambda {\left( v\right) }$$.

Similar to the BIT operator case, this correspondence is one-to-one^16^.

We need a metric on $V^{\textrm{res}}$ to explain the condition of reservoirs for universality proposed in^16^. Using $w:{\mathbb {R}}_+\rightarrow \left( 0,1 \right]$ in Eq. (10), we define the weighted norm $\left\| v \right\| _w$ of $v:\left[ -t,0 \right] \rightarrow {\mathbb {R}}^n$ as

23 $$\begin{aligned} \left\| v \right\| _w = \sup _{\tau \in \left[ {-t,0} \right] } \left\| v{\left( \tau \right) }\right\| w{\left( -\tau \right) }. \end{aligned}$$

Let $\theta :{\mathbb {R}}_+\rightarrow {\mathbb {R}}_+$ be a strictly increasing, bounded, and continuous function. For *w* and $\theta$, we define the distance $d{\left( {v_1},{v_2} \right) }$ between $v_1$, $v_2\in V^{\textrm{res}}$ as

24 $$\begin{aligned} d{\left( {v_1},{v_2} \right) }=\left\| v_1^{\left[ t_{\min }\right] }-v_2^{\left[ t_{\min }\right] } \right\| _w+\left| \theta {\left( t_1 \right) }-\theta {\left( t_2 \right) }\right| , \end{aligned}$$

where $t_1=\lambda {\left( v_1\right) }$, $t_2=\lambda {\left( v_2\right) }$, and $t_{\min }=\min \left\{ t_1,t_2 \right\}$. See Assumption 1 in^16^ for detailed conditions for the map $d:V^{\textrm{res}}\times V^{\textrm{res}}\rightarrow {\mathbb {R}}_+$ to be a metric. We extend *d* onto $V^{\textrm{res}}\cup V$ by defining $\lambda {\left( v\right) }=\infty$, $v^{\left[ \infty \right] }=v$, and $\theta {\left( \infty \right) }=\lim _{t\rightarrow \infty }\theta {\left( t\right) }$ for any $v\in V$. With this extension, the metrics in Eqs. (11) and (24) are equivalent on *V*. Hereafter, we use *d* as a metric on $V^{\textrm{res}}\cup V$. Any $v\in V$ is an accumulation point of $V^{\textrm{res}}$ because $v^{\left[ t\right] }\in V^{\textrm{res}}$ converges to *v* as $t\rightarrow \infty$. As shown in Proposition 5 in^16^, $V^{\textrm{res}}\cup V$ is compact. Therefore, $V^{\textrm{res}}\cup V=\overline{V^{\textrm{res}}}$ holds, where $\overline{\;\cdot \;}$ means closure. In^16^, the following condition is proposed for a reservoir to be universal.

#### Definition 9

Let ${\mathbb {F}}$ be a set of causal RIT operators $F_1,\ldots ,F_m$ and let $f_1,\ldots ,f_m:V^{\textrm{res}}\rightarrow {\mathbb {R}}$ correspond to each operator in ${\mathbb {F}}$. The set ${\mathbb {F}}$ is said to have the neighborhood separation property (NSP) if the following holds for any distinct $v_1$, $v_2\in \overline{V^{\textrm{res}}}$:

25 $$\begin{aligned} \exists \delta >0,\overline{\left( f_1,\ldots ,f_m\right) {\left( N_\delta {\left( v_1 \right) }\cap V^{\textrm{res}} \right) }}\cap \overline{\left( f_1,\ldots ,f_m\right) {\left( N_\delta {\left( v_2 \right) }\cap V^{\textrm{res}}\right) }}=\emptyset , \end{aligned}$$

where $N_\delta {\left( v \right) }$ is a $\delta$-neighborhood of $v\in \overline{V^{\textrm{res}}}$ defined as follows:

26 $$\begin{aligned} N_\delta {\left( v \right) }=\left\{ v'\in \overline{V^{\textrm{res}}}\left| d{\left( v',v\right) }<\delta \right. \right\} . \end{aligned}$$

The NSP guarantees that the images of neighborhoods of distinct points are disjoint. Hence, we can say that the NSP is the “strong injectivity” of the map $\left( f_1,\ldots ,f_m\right)$.

In^16^, target operator $F^*:U^R\rightarrow Y^R$ is assumed to correspond to a uniformly continuous functional. As shown in Proposition 8 in^16^, this assumption gives $F^*$ three properties. The first is the following:

27 $$\begin{aligned} \forall \varepsilon>0,\forall u_1\in U^R,\forall t\ge 0,\exists \delta >0,\forall u_2\in U^R,\left[ \max _{\tau \in \left[ 0,t\right] }\left\| u_1{\left( \tau \right) }-u_2{\left( \tau \right) }\right\|<\delta \Rightarrow \left| F^*u_1{\left( t\right) }-F^*u_2{\left( t\right) }\right| <\varepsilon \right] . \end{aligned}$$

Roughly speaking, this is a “continuity” of the output value with respect to past inputs. Hence, $F^*$ is also causal. The second property is the equicontinuity of output signals, that is,

28 $$\begin{aligned} \forall \varepsilon>0,\forall t\ge 0,\exists \delta >0,\forall y\in F^*{\left( U\right) },\forall t'\ge 0,\left[ \left| t-t'\right|<\delta \Rightarrow \left| y{\left( t\right) }-y{\left( t'\right) }\right| <\varepsilon \right] . \end{aligned}$$

The third property is fading memory of an RIT operator^16^, which means that older inputs have less influence on the present output. At first glance, the uniform continuity of the target’s corresponding functional is a stricter condition than that in^2^. However, in^2^, continuity implies uniform continuity because the functional domain *V* is compact. Hence, no difference exists between the two.

The result in^16^ is described as follows:

#### Theorem 3

(Sugiura et al.^16^) Let ${\mathbb {F}}^*$ be the set of RIT operators corresponding to a uniformly continuous functional and let reservoir ${\mathbb {F}}$ be a set of bounded RIT operators. Suppose that ${\mathbb {F}}$ has the NSP. Then, reservoir ${\mathbb {F}}$ is universal for uniform approximations in ${\mathbb {F}}^*$.

Operator $F:U^R\rightarrow Y^R$ is said to be bounded if $F{\left( U^R\right) }$ is uniformly bounded, that is,

29 $$\begin{aligned} \exists c\ge 0,\forall y\in F{\left( U\right) },\forall t\ge 0,\left| y{\left( t\right) }\right| \le c. \end{aligned}$$

To summarize the result in^16^, a reservoir with the NSP that contains only bounded operators is universal.

### Existence of universal finite reservoir

Using Theorem 3, we show that a universal reservoir ${\mathbb {F}}$ with a single output exists. If the reservoir ${\mathbb {F}}$ is universal, we can easily construct a universal reservoir with a higher dimensional output by adding arbitrary operators to ${\mathbb {F}}$. Hence, the universality of ${\mathbb {F}}$ means that, for any dimension, a universal reservoir exists with the output of that specified dimension. Let operator $F:U^R\rightarrow Y^R$ and functional $f:V^{\textrm{res}}\rightarrow {\mathbb {R}}$ correspond to each other. Then, the NSP of reservoir ${\mathbb {F}}=\left\{ F\right\}$ is written as follows:

30 $$\begin{aligned} \forall v_1,v_2\in \overline{V^{\textrm{res}}},\left[ v_1\ne v_2\Rightarrow \left( \exists \delta >0,\overline{f{\left( N_\delta {\left( v_1 \right) }\cap V^{\textrm{res}} \right) }}\cap \overline{f{\left( N_\delta {\left( v_2 \right) }\cap V^{\textrm{res}}\right) }}=\emptyset \right) \right] . \end{aligned}$$

The following proposition provides a stronger but simpler condition than Eq. (30):

#### Proposition 3

Let $f:V^{\textrm{res}}\rightarrow {\mathbb {R}}$. Suppose that there is a continuous left inverse $g:\overline{f{\left( V^{\textrm{res}}\right) }}\rightarrow \overline{V^{\textrm{res}}}$ of *f*, that is, a continuous map *g* that satisfies the following:

31 $$\begin{aligned} g\circ f=\textrm{id}_{V^{\textrm{res}}}. \end{aligned}$$

Then, *f* satisfies Eq. (30).

#### Proof of Proposition 3

We prove the contraposition. Suppose that there are distinct $v_1$, $v_2\in \overline{V^{\textrm{res}}}$ that satisfy the following:

32 $$\begin{aligned} \forall \delta >0,\overline{f{\left( V_1 \right) }}\cap \overline{f{\left( V_2\right) }}\ne \emptyset , \end{aligned}$$

where $V_1$, $V_2\subset V^{\textrm{res}}$ are defined as follows:

33 $$\begin{aligned} V_1=N_\delta {\left( v_1 \right) }\cap V^{\textrm{res}},\quad V_2=N_\delta {\left( v_2 \right) }\cap V^{\textrm{res}}. \end{aligned}$$

Because of $v_1\ne v_2$, we obtain $\overline{V_1}\cap \overline{V_2}=\emptyset$ by setting $\delta =d{\left( v_1,v_2\right) }/3$. Let $\left( \alpha _{1,i}\right) _{i\in {\mathbb {N}}}\in f{\left( V_1\right) }$ and $\left( \alpha _{2,i}\right) _{i\in {\mathbb {N}}}\in f{\left( V_2\right) }$ be sequences that converge to $\alpha \in \overline{f{\left( V_1 \right) }}\cap \overline{f{\left( V_2\right) }}$. Suppose that $g:\overline{f{\left( V^{\textrm{res}}\right) }}\rightarrow \overline{V^{\textrm{res}}}$ is a left inverse of *f*. Then, $g{\left( \alpha _{1,i}\right) }$ and $g{\left( \alpha _{2,i}\right) }$ are contained in $V_1$ and $V_2$, respectively. Because of $\overline{V_1}\cap \overline{V_2}=\emptyset$, $g{\left( \alpha _{1,i}\right) }$ and $g{\left( \alpha _{2,i}\right) }$ do not converge to the same point as $i\rightarrow \infty$, even though $\alpha _{1,i}$ and $\alpha _{2,i}$ converge to $\alpha$. Hence, *g* is not continuous at $\alpha \in \overline{f{\left( V^{\textrm{res}}\right) }}$. $\square$

To prove that a functional $f:V^{\textrm{res}}\rightarrow {\mathbb {R}}$ with a continuous left inverse exists, we use the following theorem.

#### Theorem 4

(Hahn–Mazurkiewicz theorem^20^) Let *E* be a connected, locally connected, and compact metric space. Then, a continuous surjection from $\left[ 0,1\right]$ to *E* exists.

Metric space $\overline{V^{\textrm{res}}}=V^{\textrm{res}}\cup V$ is compact^16^. As we show later in this paper, $\overline{V^{\textrm{res}}}$ is connected and locally connected. Hence, from Theorem 4, a space-filling curve $g:\left[ 0,1\right] \rightarrow \overline{V^{\textrm{res}}}$ exists, which is continuous and surjective. Using the axiom of choice, we obtain a functional $f:V^{\textrm{res}}\rightarrow {\mathbb {R}}$ with a continuous left inverse as a right inverse of *g*. Therefore, a reservoir that contains only one operator can have the NSP. To prove that $\overline{V^{\textrm{res}}}$ is connected and locally connected, we assume that the set $A\subset {\mathbb {R}}^n$ in Eq. (8) is convex.

#### Proposition 4

The set $\overline{V^{\textrm{res}}}$ is connected.

#### Proof of Proposition 4

We prove that $\overline{V^{{\textrm{res}}}}$ is arcwise connected, which is a stronger condition than being connected. Let $v_1$ and $v_2$ be arbitrary inputs in $\overline{V^{{\textrm{res}}}}$. The set $\overline{V^{\textrm{res}}}$ is said to be arcwise connected if there is some continuous map $P:\left[ 0,1\right] \rightarrow \overline{V^{{\textrm{res}}}}$ that satisfies $P{\left( 0\right) }=v_1$ and $P{\left( 1\right) }=v_2$. Such a map *P* is called a path from $v_1$ to $v_2$. Let $t_1=\lambda {\left( v_1\right) }$ and $t_2=\lambda {\left( v_2\right) }$. If a path exists from $v_1$ to $v_2$, one exists from $v_2$ to $v_1$. Hence, we can assume that $t_1\le t_2$ without loss of generality. Let $v_3=v_2^{\left[ t_1\right] }$, that is, $v_3$ has the same domain as $v_1$ and the same value as $v_2$. We form a path from $v_1$ to $v_2$ through $v_3$.

First, we form a path from $v_1$ to $v_3$. Because $v_1$ and $v_3$ has the same domain $\left[ -t_1,0\right]$, we can define a map $P_1:\left[ 0,1\right] \rightarrow \overline{V^{\textrm{res}}}$ as follows:

34 $$\begin{aligned} P_1{\left( \alpha \right) }=\left( 1-\alpha \right) v_1+\alpha v_3\quad \left( \alpha \in \left[ 0,1\right] \right) . \end{aligned}$$

Map $P_1$ satisfies $P_1{\left( 0\right) }=v_1$ and $P_1{\left( 1\right) }=v_3$. Because the input range $A\subset {\mathbb {R}}^n$ is convex, the image of $P_1$ is included in $\overline{V^{\textrm{res}}}$. Map $P_1$ is continuous because, for any $\alpha _1$, $\alpha _2\in \left[ 0,1\right]$, the distance between $P_1{\left( \alpha _1\right) }$ and $P_1{\left( \alpha _2\right) }$ is formulated as follows:

35 $$\begin{aligned} d{\left( P_1{\left( \alpha _1\right) },P_1{\left( \alpha _2\right) }\right) }=\left| \alpha _1-\alpha _2\right| \left\| v_1-v_3\right\| _w. \end{aligned}$$

Therefore, map $P_1$ is a path from $v_1$ to $v_3$.

Second, we form a path from $v_3$ to $v_2$. The function $\theta$ has inverse $\theta ^{-1}$ because it is strictly increasing and continuous. Using $\theta ^{-1}$, we define map $\mu :\left[ 0,1\right] \rightarrow \left[ t_1,t_2\right]$ as follows:

36 $$\begin{aligned} \mu {\left( \alpha \right) }=\theta ^{-1}{\left( \left( 1-\alpha \right) \theta {\left( t_1\right) }+\alpha \theta {\left( t_2\right) }\right) }\quad \left( \alpha \in \left[ 0,1\right] \right) , \end{aligned}$$

where $\theta {\left( \infty \right) }=\lim _{t\rightarrow \infty }\theta {\left( t\right) }$ and $\theta ^{-1}{\left( \theta {\left( \infty \right) }\right) }=\infty$. We define a map $P_2:\left[ 0,1\right] \rightarrow \overline{V^{\textrm{res}}}$ as follows:

37 $$\begin{aligned} P_2{\left( \alpha \right) }=v_2^{\left[ \mu {\left( \alpha \right) }\right] }\quad \left( \alpha \in \left[ 0,1\right] \right) . \end{aligned}$$

Map $P_2$ satisfies $P_2{\left( 0\right) }=v_3$ and $P_2{\left( 1\right) }=v_2$ because $\mu {\left( 0\right) }=t_1$ and $\mu {\left( 1\right) }=t_2$ hold. From the definition (21) of $V^{\textrm{res}}$, the image of $P_2$ is included in $\overline{V^{\textrm{res}}}$. Map $P_2$ is continuous because, for any $\alpha _1$, $\alpha _2\in \left[ 0,1\right]$, the distance between $P_2{\left( \alpha _1\right) }$ and $P_2{\left( \alpha _2\right) }$ is formulated as follows:

38 $$\begin{aligned} d{\left( P_2{\left( \alpha _1\right) },P_2{\left( \alpha _2\right) }\right) }=\left| \theta {\left( \mu {\left( \alpha _1\right) }\right) }-\theta {\left( \mu {\left( \alpha _2\right) }\right) }\right| =\left| \alpha _1-\alpha _2\right| \left| \theta {\left( t_1\right) }-\theta {\left( t_2\right) }\right| . \end{aligned}$$

Therefore, map $P_2$ is a path from $v_3$ to $v_2$.

Using $P_1$ and $P_2$, we define path $P:\left[ 0,1\right] \rightarrow \overline{V^{\textrm{res}}}$ from $v_1$ to $v_2$ as follows:

39 $$\begin{aligned} P{\left( \alpha \right) }= {\left\{ \begin{array}{ll} P_1{\left( 2\alpha \right) }&{}\left( 0\le \alpha \le \frac{1}{2}\right) \\ P_2{\left( 2\alpha -1\right) }&{}\left( \frac{1}{2}<\alpha \le 1\right) \end{array}\right. }. \end{aligned}$$

Therefore, the set $\overline{V^{\textrm{res}}}$ is arcwise connected. $\square$

To prove that the set $\overline{V^{\textrm{res}}}$ is locally connected, we use the following proposition:

#### Proposition 5

For any $v_1$, $v_2\in \overline{V^{\textrm{res}}}$, path $P:\left[ 0,1\right] \rightarrow \overline{V^{\textrm{res}}}$ from $v_1$ to $v_2$ exists that satisfies the following:

40 $$\begin{aligned} \forall \alpha \in \left[ 0,1\right] ,d{\left( v_1,v_2\right) }=d{\left( v_1,P{\left( \alpha \right) }\right) }+d{\left( P{\left( \alpha \right) },v_2\right) }. \end{aligned}$$

Equation (40) means that path *P* is “the shortest” between $v_1$ and $v_2$, that is, equality holds in the triangle inequality between any point on the path and the two endpoints.

#### Proof of Proposition 5

Let $v_1$ and $v_2$ be arbitrary inputs in $\overline{V^{\textrm{res}}}$. If path *P* from $v_1$ to $v_2$ exists that satisfies Eq. (40), a similar path from $v_2$ to $v_1$ exists. Hence, for $t_1=\lambda {\left( v_1\right) }$ and $t_2=\lambda {\left( v_2\right) }$, we can assume that $t_1\le t_2$ without loss of generality. We show that path $P:\left[ 0,1\right] \rightarrow \overline{V^{\textrm{res}}}$ defined by (39) satisfies Eq. (40) using the following three lemmas: $\square$

#### Lemma 2

Path $P_1:\left[ 0,1\right] \rightarrow \overline{V^{\textrm{res}}}$ defined by Eq. (34) satisfies the following:

41 $$\begin{aligned} \forall \alpha \in \left[ 0,1\right] ,d{\left( v_1,v_3\right) }=d{\left( v_1,P_1{\left( \alpha \right) }\right) }+d{\left( P_1{\left( \alpha \right) },v_3\right) }. \end{aligned}$$

#### Proof of Lemma 2

Using Eq. (35), distance $d{\left( v_1,v_3\right) }$ is transformed as follows:

42 $$\begin{aligned} \begin{aligned} d{\left( v_1,v_3\right) }=&\,\left\| v_1-v_3\right\| _w\\ =&\,\left| 0-\alpha \right| \left\| v_1-v_3\right\| _w+\left| \alpha -1\right| \left\| v_1-v_3\right\| _w\\ =&\,d{\left( P_1{\left( 0\right) },P_1{\left( \alpha \right) }\right) }+d{\left( P_1{\left( \alpha \right) },P_1{\left( 1\right) }\right) }\\ =&\,d{\left( v_1,P_1{\left( \alpha \right) }\right) }+d{\left( P_1{\left( \alpha \right) },v_3\right) }, \end{aligned} \end{aligned}$$

which proves Lemma 2. $\square$

#### Lemma 3

Path $P_2:\left[ 0,1\right] \rightarrow \overline{V^{\textrm{res}}}$ defined by Eq. (37) satisfies the following:

43 $$\begin{aligned} \forall \alpha \in \left[ 0,1\right] ,d{\left( v_3,v_2\right) }=d{\left( v_3,P_2{\left( \alpha \right) }\right) }+d{\left( P_2{\left( \alpha \right) },v_2\right) }. \end{aligned}$$

#### Proof of Lemma 3

Using Eq. (38), distance $d{\left( v_3,v_2\right) }$ is transformed as follows:

44 $$\begin{aligned} \begin{aligned} d{\left( v_3,v_2\right) }=&\,\left| \theta {\left( t_1\right) }-\theta {\left( t_2\right) }\right| \\ =&\,\left| 0-\alpha \right| \left| \theta {\left( t_1\right) }-\theta {\left( t_2\right) }\right| +\left| \alpha -1\right| \left| \theta {\left( t_1\right) }-\theta {\left( t_2\right) }\right| \\ =&\,d{\left( P_2{\left( 0\right) },P_2{\left( \alpha \right) }\right) }+d{\left( P_2{\left( \alpha \right) },P_2{\left( 1\right) }\right) }\\ =&\,d{\left( v_3,P_2{\left( \alpha \right) }\right) }+d{\left( P_2{\left( \alpha \right) },v_2\right) }, \end{aligned} \end{aligned}$$

which proves Lemma 3. $\square$

#### Lemma 4

The input $v_3=v_2^{\left[ t_1\right] }$ satisfies the following:

45 $$\begin{aligned} d{\left( v_1,v_2\right) }=d{\left( v_1,v_3\right) }+d{\left( v_3,v_2\right) }. \end{aligned}$$

#### Proof of Lemma 4

Distance $d{\left( v_1,v_2\right) }$ is transformed as follows:

46 $$\begin{aligned} \begin{aligned} d{\left( v_1,v_2\right) }=&\,\left\| v_1-v_2^{\left[ t_1\right] }\right\| _w+\left| \theta {\left( t_1\right) }-\theta {\left( t_2\right) }\right| \\ =&\,\left\| v_1-v_3\right\| _w+\left| \theta {\left( \lambda {\left( v_3\right) }\right) }-\theta {\left( t_2\right) }\right| \\ =&\,d{\left( v_1,v_3\right) }+d{\left( v_3,v_2\right) }, \end{aligned} \end{aligned}$$

which proves Lemma 4. $\square$

Let $\alpha$ be an arbitrary number in $\left[ 0,1\right]$. We show that the following holds:

47 $$\begin{aligned} d{\left( v_1,P{\left( \alpha \right) }\right) }+d{\left( P{\left( \alpha \right) },v_2\right) }\le d{\left( v_1,v_2\right) }. \end{aligned}$$

If $0\le \alpha \le \frac{1}{2}$, we obtain Eq. (47) as follows:

48 $$\begin{aligned} d{\left( v_1,P{\left( \alpha \right) }\right) }+d{\left( P{\left( \alpha \right) },v_2\right) }\le d{\left( v_1,P{\left( \alpha \right) }\right) }+d{\left( P{\left( \alpha \right) },v_3\right) }+d{\left( v_3,v_2\right) } = d{\left( v_1,v_3\right) }+d{\left( v_3,v_2\right) } =d{\left( v_1,v_2\right) }. \end{aligned}$$

The inequality is a triangle inequality. The first equality is obtained from Lemma 2 because $P{\left( \alpha \right) }=P_1{\left( 2\alpha \right) }$ holds. The second equality is obtained from Lemma 4. If $\frac{1}{2}<\alpha \le 1$, we obtain Eq. (47) as follows:

49 $$\begin{aligned} d{\left( v_1,P{\left( \alpha \right) }\right) }+d{\left( P{\left( \alpha \right) },v_2\right) }\le d{\left( v_1,v_3\right) }+d{\left( v_3,P{\left( \alpha \right) }\right) }+d{\left( P{\left( \alpha \right) },v_2\right) } =d{\left( v_1,v_3\right) }+d{\left( v_3,v_2\right) } =d{\left( v_1,v_2\right) }. \end{aligned}$$

The inequality is a triangle inequality. The first equality is obtained from Lemma 3 because $P{\left( \alpha \right) }=P_2{\left( 2\alpha -1\right) }$ holds. The second equality is obtained from Lemma 4. From Eq. (47) and the triangle inequality $d{\left( v_1,v_2\right) }\le d{\left( v_1,P{\left( \alpha \right) }\right) }+d{\left( P{\left( \alpha \right) },v_2\right) }$, we obtain Eq. (40), which proves Proposition 5. $\square$

Using Proposition 5, we prove the following proposition:

#### Proposition 6

The set $\overline{V^{\textrm{res}}}$ is locally connected.

#### Proof of Proposition 6

We prove that $\overline{V^{\textrm{res}}}$ is locally arcwise connected, which is a stronger condition than being locally connected. Let *v* be an arbitrary input in $\overline{V^{\textrm{res}}}$. The set $\overline{V^{\textrm{res}}}$ is said to be locally arcwise connected if, for any open set $V_1\subset \overline{V^{\textrm{res}}}$ including *v*, an arcwise connected open set $V_2\subset V_1$ exists that includes *v*. Therefore, it is sufficient for the Proof of Proposition 6 to show that a $\delta$-neighborhood $N_\delta {\left( v\right) }\subset \overline{V^{\textrm{res}}}$ of *v* is arcwise connected for any $\delta >0$. Let $\delta$ be an arbitrary positive number and $v_1$, $v_2$ be arbitrary inputs in $N_\delta {\left( v\right) }$. From Proposition 5 and $d{\left( v,v_1\right) }<\delta$, path $P_1:\left[ 0,1\right] \rightarrow \overline{V^{\textrm{res}}}$ from $v_1$ to *v* exists that satisfies the following for any $\alpha \in \left[ 0,1\right]$:

50 $$\begin{aligned} d{\left( v,P_1{\left( \alpha \right) }\right) }=d{\left( v,v_1\right) }-d{\left( P_1{\left( \alpha \right) },v_1\right) }<\delta -d{\left( P_1{\left( \alpha \right) },v_1\right) }\le \delta . \end{aligned}$$

Hence, $P_1{\left( \left[ 0,1\right] \right) }\subset N_\delta {\left( v\right) }$ holds. Similarly, we obtain path $P_2:\left[ 0,1\right] \rightarrow N_\delta {\left( v\right) }$ from *v* to $v_2$. Therefore, path $P:\left[ 0,1\right] \rightarrow N_\delta {\left( v\right) }$ from $v_1$ to $v_2$ exists, which proves Proposition 6. $\square$

Using Theorem 3 and Propositions 3, 4, and 6, we show that a universal reservoir exists that has only one output.

#### Theorem 5

Let ${\mathbb {F}}^*$ be the set of RIT operators corresponding to a uniformly continuous functional. Assume the axiom of choice and that the set $A\subset {\mathbb {R}}^n$ in Eq. (8) is convex. Then, reservoir ${\mathbb {F}}$ exists that is a set of only one RIT operator and ${\mathbb {F}}$ is universal for uniform approximations in ${\mathbb {F}}^*$.

#### Proof of Theorem 5

Metric space $\overline{V^{\textrm{res}}}=V^{\textrm{res}}\cup V$ is compact^16^. From Propositions 4 and 6, $\overline{V^{\textrm{res}}}$ is connected and locally connected. Hence, from Theorem 4, a continuous surjection $g:\left[ 0,1\right] \rightarrow \overline{V^{\textrm{res}}}$ exists. We define a set $S\subset \left[ 0,1\right]$ as follows:

51 $$\begin{aligned} S=\left\{ \alpha \in \left[ 0,1\right] \left| g{\left( \alpha \right) }\in V^{\textrm{res}}\right. \right\} . \end{aligned}$$

Let $g|_S:S\rightarrow V^{\textrm{res}}$ be a restriction of *g* to *S*. Because $g|_S$ is a surjection to $V^{\textrm{res}}$, the axiom of choice provides a right inverse $f:V^{\textrm{res}}\rightarrow S$ of $g|_S$, that is, *f* satisfies $g|_S\circ f=\textrm{id}_{V^{\textrm{res}}}$. Functional *f* and an another restriction $g|_{\overline{S}}:\overline{S}\rightarrow \overline{V^{\textrm{res}}}$ of *g* also satisfy $g|_{\overline{S}}\circ f=\textrm{id}_{V^{\textrm{res}}}$. Because the restriction $g|_{\overline{S}}$ is continuous, from Proposition 3, *f* satisfies Eq. (30). Hence, reservoir ${\mathbb {F}}=\left\{ F\right\}$ has the NSP, where $F:U^R\rightarrow Y^R$ is the corresponding operator of *f*. Because of $S\subset \left[ 0,1\right]$, functional *f* and operator *F* are bounded. Therefore, reservoir ${\mathbb {F}}$ satisfies the condition of Theorem 3 and is universal for uniform approximations in ${\mathbb {F}}^*$. $\square$

Theorem 5 shows that a universal finite reservoir exists, which achieves the low computational cost of training. Moreover, a universal reservoir exists independent of the dimension of its output. The reservoir obtained from a right inverse of a space-filling curve proposed in the Proof of Theorem 5 is probably chaotic. This result suggests that chaos is key to the reservoir’s universality. In practice, chaotic reservoirs have already been considered in research and are known to be useful^21,22^.

The difference between the results of Sections “Reservoir computing represented by bi-infinite-time operators” and “Reservoir computing represented by right-infinite-time operators” is not caused by the difference between the BIT and RIT operators but the difference between the conditions of functionals $f_1,\ldots ,f_m$ corresponding to operators in the reservoir. Fading memory and the separation property, which are the conditions of Theorem 1, require the map $\left( f_1,\ldots ,f_m\right)$ to be continuous and injective; however, this is impossible. By contrast, the NSP, which is the condition of Theorem 3, only requires the “strong injectivity” of $\left( f_1,\ldots ,f_m\right)$, which is possible as described thus far. The NSP is also defined for a reservoir represented by BIT operators as follows:

#### Definition 10

Let ${\mathbb {F}}$ be a set of causal BIT operators $F_1,\ldots ,F_m$ and let $f_1,\ldots ,f_m:V\rightarrow {\mathbb {R}}$ correspond to each operator in ${\mathbb {F}}$. The set ${\mathbb {F}}$ is said to have the NSP if the following holds for any distinct $v_1$, $v_2\in V$:

52 $$\begin{aligned} \exists \delta >0,\overline{\left( f_1,\ldots ,f_m\right) {\left( N_\delta {\left( v_1 \right) }\right) }}\cap \overline{\left( f_1,\ldots ,f_m\right) {\left( N_\delta {\left( v_2 \right) }\right) }}=\emptyset , \end{aligned}$$

where $N_\delta {\left( v \right) }\subset V$ is a $\delta$-neighborhood of $v\in V$.

A finite reservoir with the NSP of Definition 10 can be obtained in the same manner as in the RIT operator case. Using Theorem 4 in^16^, that is, an extension of the Stone–Weierstrass theorem, we can derive the universality of Definition 1 from the NSP of Definition 10. Therefore, the NSP is the essence of proving the existence of a universal finite reservoir.

## Conclusion

We discussed whether a universal finite reservoir exists under the assumption that the readout is a polynomial and the target operator has fading memory. In the discussion, we considered two sufficient conditions for the reservoir to be universal proposed by Maass et al.^2^ and in^16^. First, we showed that no finite reservoir satisfies the condition in^2^. Supposing that such a reservoir exists, we derived the contradiction that the input function space and a subset of finite-dimensional vector space are homeomorphic. Next, we proposed an example of a universal reservoir that has a single output and the NSP, which is the condition in^16^. The functional corresponding to the operator representing the reservoir has a continuous left inverse. We showed that this is a stronger condition than the NSP. The functional is defined as a right inverse of the continuous surjection from $\left[ 0,1\right]$ to the input space of the functional. The surjection is given by the Hahn–Mazurkiewicz theorem. Our example means that, for any dimension, a universal reservoir exists that has the output of that specified dimension. This result is particularly important for the use of physical reservoirs, which are difficult to train.

### Supplementary Information

Supplementary Information.

## Data Availability

All data generated or analyzed during this study are included in this published article and its supplementary information files.
