# Supplementary material for: Existence of reservoir with finite-dimensional output for universal reservoir computing
Source: Sci Rep. 2024 Apr 11;14:8448. doi: 10.1038/s41598-024-56742-7 (PMC11006892; doi:10.1038/s41598-024-56742-7)
Supplement: Supplementary file 1 — Supplementary Information. [file 41598_2024_56742_MOESM1_ESM.pdf]

# Supplementary Material of "Existence of Reservoir with Finite-Dimensional Output for Universal Reservoir Computing"

**Shuhei Sugiura[1], Ryo Ariizumi[2], Toru Asai[1], and Shun-ichi Azuma[3]**

[1]Nagoya University, Graduate School of Engineering, Nagoya, 464-8603, Japan
[2]Tokyo University of Agriculture and Technology, Department of Mechanical System Engineering, Tokyo, 183-0057, Japan
[3]Kyoto University, Graduate School of Informatics, Kyoto, 606-8501, Japan

## ABSTRACT

In this document, we provide the proofs of Propositions 1 and 2.

## 1 Proof of Proposition 1

First, we prove that functional $f$ is continuous if operator $F$ has fading memory. Let $v_1 \in V$ be an arbitrary input and $\varepsilon$ be an arbitrary positive number. Because $F$ has fading memory, $\delta > 0$ and $T > 0$ exist that satisfy the following for any $v_2 \in V$:

$$\max_{\tau\in[-T,0]} \|u_1(\tau) - u_2(\tau)\| < \delta \Rightarrow |Fu_1(0) - Fu_2(0)| < \varepsilon, \tag{1}$$

where $u_1, u_2 \in U^B$ are defined as follows:

$$u_1(\tau) = \begin{cases} v_1(\tau) & (\tau \le 0) \\ v_1(0) & (\tau > 0) \end{cases}, \quad u_2(\tau) = \begin{cases} v_2(\tau) & (\tau \le 0) \\ v_2(0) & (\tau > 0) \end{cases}. \tag{2}$$

Suppose that $v_2 \in V$ satisfies $d(v_1, v_2) < w(T)\delta$. Because function $w$ is non-increasing, we obtain

$$\max_{\tau\in[-T,0]} \|v_1(\tau) - v_2(\tau)\| \le \sup_{\tau\in[-T,0]} \|v_1(\tau) - v_2(\tau)\| \frac{w(-\tau)}{w(T)} \le \frac{d(v_1, v_2)}{w(T)} < \delta. \tag{3}$$

From Eqs. (2) and (3), we have $\max_{\tau\in[-T,0]} \|u_1(\tau) - u_2(\tau)\| < \delta$. Hence, we obtain $|Fu_1(0) - Fu_2(0)| < \varepsilon$ from Eq. (1). Because $F$ and $f$ correspond to each other, we obtain $|f(v_1) - f(v_2)| < \varepsilon$. Therefore, $f$ is continuous at $v_1 \in V$.

Next, we prove that operator $F$ has fading memory if functional $f$ is continuous. Let $u_1 \in U^B$ be an arbitrary input and $\varepsilon$ be an arbitrary positive number. Because $f$ is continuous, $\delta > 0$ exists that satisfies the following for any $u_2 \in U^B$:

$$d(v_1, v_2) < \delta \Rightarrow |f(v_1) - f(v_2)| < \varepsilon, \tag{4}$$

where $v_1, v_2 \in V$ are restrictions of $u_1, u_2$ onto $\mathbb{R}_-$ defined as follows:

$$v_1(\tau) = u_1(\tau), \quad v_2(\tau) = u_2(\tau) \qquad (\tau \le 0). \tag{5}$$

Because $w(t)$ converges to 0 as $t \to \infty$, $T > 0$ exists that satisfies $Mw(T) < \delta$, where $M \ge 0$ is the maximum difference between the two input values, that is,

$$M = \max_{a_1, a_2 \in A} \|a_1 - a_2\|. \tag{6}$$

Suppose that $u_2 \in U^B$ satisfies the following:

$$\max_{\tau\in[-T,0]} \|u_1(\tau) - u_2(\tau)\| < \delta. \tag{7}$$

Because function $w$ is non-increasing, and its image is $(0,1]$, the following two inequalities hold from Eqs. (5) and (7):

$$\sup_{\tau\in[-T,0]} \|v_1(\tau)-v_2(\tau)\|\, w(-\tau) < \delta, \tag{8}$$

$$\sup_{\tau\le -T} \|v_1(\tau)-v_2(\tau)\|\, w(-\tau) \le Mw(T) < \delta. \tag{9}$$

These two inequalities mean that $d(v_1,v_2) < \delta$. Hence, we obtain $|f(v_1)-f(v_2)| < \varepsilon$ from Eq. (4). Because $F$ and $f$ correspond to each other, we obtain $|Fu_1(0)-Fu_2(0)| < \varepsilon$. Therefore, $F$ has fading memory.

## 2 Proof of Proposition 2

First, we prove that if $\mathbb{F}$ has the separation property, $(f_1,\ldots,f_m)$ is injective. Let $v_1$, $v_2 \in V$ be arbitrary distinct inputs. We define $u_1$, $u_2 \in U^B$ using Eq. (2), and $\tau \le 0$ exists that satisfies $u_1(\tau) \ne u_2(\tau)$. Because $\mathbb{F}$ has the separation property, $i \in \{1,\ldots,m\}$ exists that satisfies $F_i u_1(0) \ne F_i u_2(0)$. Because $F_i$ and $f_i$ correspond to each other, we obtain $f_i(v_1) \ne f_i(v_2)$. Therefore, $(f_1,\ldots,f_m)$ is injective.

Next, we prove that if $(f_1,\ldots,f_m)$ is injective, $\mathbb{F}$ has the separation property. Let $u_1$, $u_2 \in U^B$ be arbitrary inputs such that there is some $\tau \le 0$ that satisfies $u_1(\tau) \ne u_2(\tau)$. We define $v_1$, $v_2 \in V$ using Eq. (5), and $v_1 \ne v_2$ holds. Because $(f_1,\ldots,f_m)$ is injective, $i \in \{1,\ldots,m\}$ exists that satisfies $f_i(v_1) \ne f_i(v_2)$. Because $F_i$ and $f_i$ correspond to each other, we obtain $F_i u_1(0) \ne F_i u_2(0)$. Therefore, $\mathbb{F}$ has the separation property.
